# HGF and IL-10 expressing ALB::GFP reporter cells generated from iPSCs show robust anti-fibrotic property in acute fibrotic liver model

**DOI:** 10.1186/s13287-020-01745-0

**Published:** 2020-08-03

**Authors:** Ja Sung Choi, In Sil Jeong, Young-Jin Park, Sung-Whan Kim

**Affiliations:** 1 Department of Internal Medicine, Catholic Kwandong University College of Medicine, International St. Mary’s Hospital, Incheon, Republic of Korea; 2 Department Medicine, Catholic Kwandong University College of Medicine, Gangneung, Republic of Korea; 3 Department of Family Medicine, Dong-A University College of Medicine, Dong-A University Medical Center, Busan, Republic of Korea; 4 International St. Mary’s Hospital, 25, Simgok-ro 100 beon-gil, Seo-gu, Incheon, 404-190 South Korea

**Keywords:** Albumin, Cell therapy, Differentiation, Gene editing, Knock-in, Reporter system, Stem cell, Hepatocyte, Liver cirrhosis, TALEN

## Abstract

**Background:**

Cell therapy using hepatocytes derived from stem cells has been regarded as a promising alternate to liver transplantation. However, the heterogeneity of these hepatocytes makes them unsuitable for therapeutic use. To overcome this limitation, we generated homogenous hepatocyte like induced hepatocyte-like (iHep) cells.

**Methods:**

iHep cells were generated from induced pluripotent stem cells (iPSCs) integrated with the albumin (ALB) reporter gene. The therapeutic properties of these iHep cells were investigated after transplantation in fibrotic liver tissues of a mouse model.

**Results:**

The iHep cells expressed hepatocyte specific genes and proteins, and exhibited high levels of hepatocyte growth factor (HGF) and interleukin (IL)-10 expressions. Transplantation of iHep cells significantly decreased thioacetamide (TAA)-induced liver fibrosis, apoptotic cells in the liver, and ameliorated abnormal liver function. Liver tissues engrafted with iHep cells exhibited decreased expression of pro-inflammatory factors such as transforming growth factor (TGF)-β, IL-6, and monocyte chemo attractant protein (MCP)-1. Furthermore, an increased number of proliferating hepatocytes and human albumin-expressing iHep cells were detected in mice liver.

**Conclusions:**

This study has investigated and proven the liver regeneration potential of genome-edited iHep cells and promises to be a strong foundation for further studies exploring cell therapy as an alternative therapeutic option for the treatment of liver fibrosis.

## Introduction

Recent studies have demonstrated that functional hepatic lineage cells can be obtained from stem cells or by direct conversion of human fibroblasts [1–9]. Differentiation of hepatocytes from different kinds of cells causes varying degrees of differentiation and cell-type specific maturation, resulting in cultures composed of heterogeneous cell types, including differentiated hepatocytes, hepatic progenitor cells, and undifferentiated stem cells. This heterogeneity results in these cells being deemed unsuitable for therapeutic use. Fusion reporter systems formed by attaching cell-specific promoters to reporter genes is an effective way for separating differentiated cells from heterogeneous cell cultures.

Lineage-specific reporter cells lines derived from embryonic stem cells (ES) or induced pluripotent stem cells (iPS) can provide optimized strategy for the direct cell differentiation protocols. Recently, knock-in reporter cell lines have been generated using transcription factors, lineage specific genes and gene editing [10–12]. The technology of gene editing using RNA-guided nucleases is very simple and enables specific gene targeting in appropriate genomic locus without random integration.

In 2017, Xie et al. demonstrated that hepatocytes capable of real-time monitoring could be produced from stem cells by adenovirus-mediated gene transfer [13]. Albumin (ALB) is the highest expressed protein in the liver, specifically expressed in mature hepatocytes. ALB promoter contains six cis-acting elements and bind to other hepatic specific factors including hatocyte nuclear factor 1 (HNF1) and D site ALB binding protein (DBP) and start transcription in the liver [14, 15]. ALB promoter has been used for the cell-specific gene delivery due to the highest activity in the liver [16]. Recently, hepatocyte cell lines, rat primary hepatocyte, bone marrow cells were transduced using adenoviral vector containing ALB promoter *ZsGreen* reporter gene [13]. However, since adenoviruses do not integrate into host genomes, their use for gene transfer resulted in transient expression of the reporter system. This limited the long-term observation of the differentiated cells.

In this study, we successfully constructed ALB reporter induced pluripotent stem cells (ALB-iPS) line using ALB::GFP (ALB promoter fused with green fluorescent protein) reporter gene and transcription activator-like effector nucleases (TALEN). In addition, we generated induced hepatocyte-like cells (iHep) derived from ALB-iPS and investigated their anti-fibrotic characteristics and therapeutic property of in liver fibrotic model.

## Materials and methods

### Cell culture

Human induced pluripotent stem cells (iPSCs) donated from National Center for Stem Cell and Regenerative Medicine in Korea. iPS cells were cultured in Essential 8™ Medium (Thermo Fisher Scientific, MA, USA) supplemented with Essential 8™ Supplement. The iPSCs culture plates were coated with vitronectin. The HepG2 cells were maintained in Dulbecco’s Modified Eagle Medium (DMEM) supplemented with 10% fetal bovine serum (FBS).

### Donor vector design

**Fig. 1:**
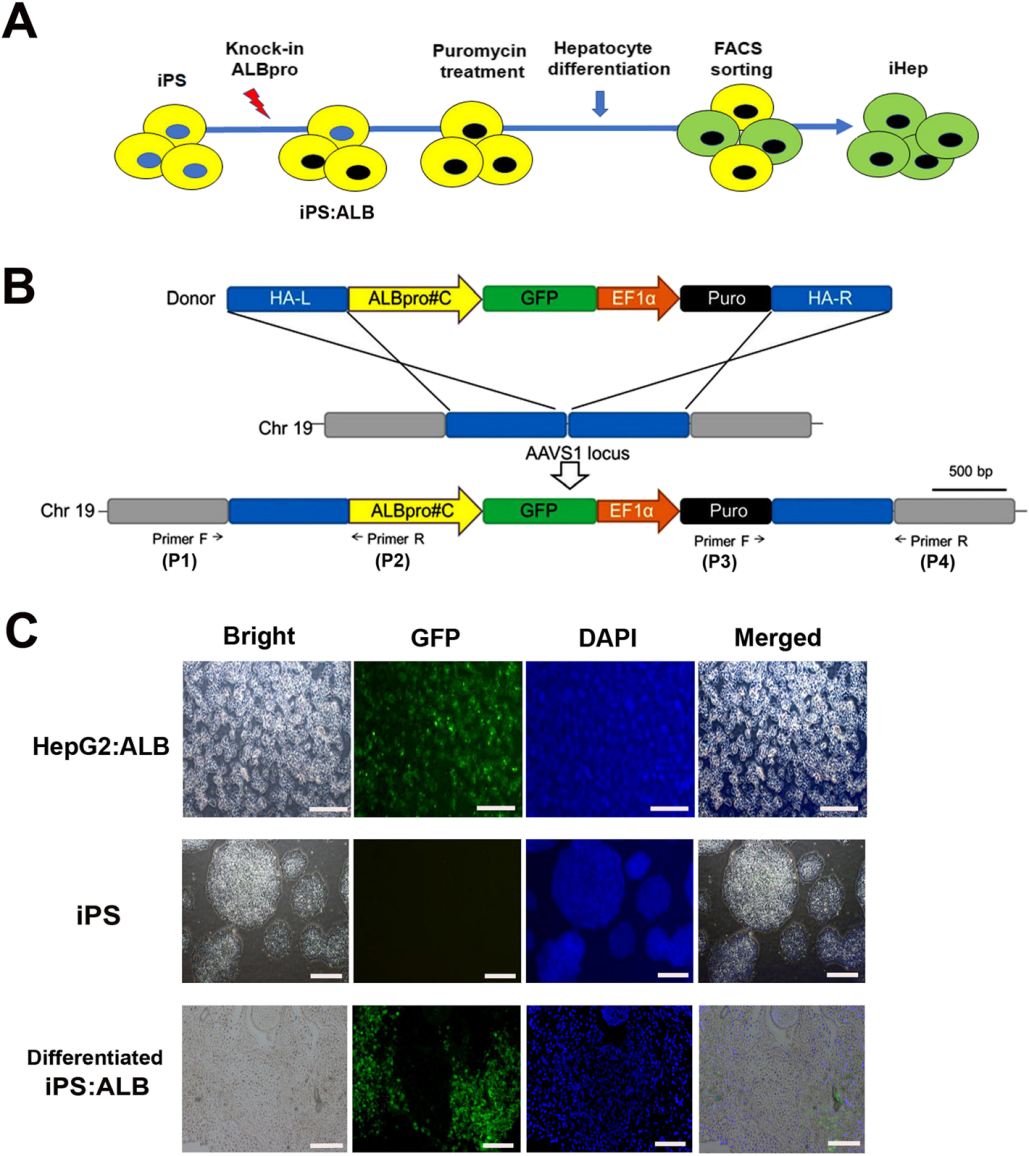
Generation of iHep cells using TALEN gene editing. **a** The protocol for the generation of iHep from iPS. Transfected iPS cells were selected after incubation with puromycin for 5 days, followed by differentiation into hepatocyte. **b** Schematic representation of the donor vector carrying the ALB promoter::GFP reporter system and DNA targeting locus of the recipient plasmid. The expression cassette containing the ALB promoter::GFP reporter and EF1α promoter-driven puromycin resistance gene was inserted into the AAVS1 site using homology-directed repair. Locations of primers for junction detection are indicated (primer F (P1, P3) and primer R (P2, P4)). Abbreviations: HA-L, left homology arm; HA-R, right homology arm; EF1α, elongation factor-1 alpha promoter; Puro, puromycin. **c** Expression of GFP in the stably transfected HepG2 and iPS. Nuclei stained with 4′-6-diamidino-2-phenylindole (DAPI,blue color). Bar = 200 μm

AAVS1 HR Donor (System Biosciences, Palo Alto, CA, USA) was modified for promoter reporter system. The PGK promoter of AAVS1 HR Donor was replaced by the ALB promoter (844 bp) and GFP reporter gene was positioned to be expressed by the ALB promoter (Fig. 1b and Supplementary Fig. 1). The GFP/puromycin of AAVS1 HR Donor was nulled and the puromycin resistance gene was cloned to be expressed by EF1 promoter.

### Transfection

Human iPS cells were maintained in Essential 8™ Medium (Thermo Fisher Scientific, MA, USA) supplemented with Essential 8™ Supplement. For electroporation, 1 × 10^5^ of human iPS cells were harvested and resuspended with 1 μg of AAVS1 left TALE-Nuclease vector (System Biosciences), AAVS1 right TALE-Nuclease vector (System Biosciences) (Supplementary Fig. 1), and ALB::GFP_AAVS1 HR Donor in 10 μL electroporation buffer; and the cells were electroporated using a Neon Transfection System (Thermo Fisher Scientific). Neon electroporation condition was 1200 Voltage, 10 width, 3 pulse 1 time.

### Puromycin selection

All experiments regarding the selection of ALB::GFP knock-in cells were performed by modifying a previous method [13]. Differentiated ALB::GFP knock-in cells were selected by incubating with 2 μg/mL puromycin for 5 days. About 30 colonies were survived and GFP expressing cells were observed from the 7th day onwards.

### Directed differentiation of genetically modified iPSCs into the hepatocyte-like cells

Hepatocytes were differentiated from iPSCs using previously described methods [4] with the following modifications*.* Briefly, the ALB::GFP iPSCs were cultured in Essential 8™ Medium with activin A (100 ng/ml) for 5 days. Following Activin A treatment, the differentiated human iPSCs were cultured in Essential 8™ Medium containing 10 ng/ml FGF2, 20 ng/ml BMP4 and 20 ng/ml HGF for 5 ~ 10 days.

### Fluorescence-activated cell sorting (FACS)

The puromycin-selected cells were harvested and washed once with phosphate buffered saline (PBS) followed by 0.05% Trypsin/EDTA treatment for cell detachment. The cells were resuspended in PBS and GFP expressing cells were sorted on the FACSAria II (BD, San Diego, CA, USA). The rate of GPF positive cells was 99.9% (Supplementary Fig. 2).

### Genomic DNA extraction and junction PCR

Genomic DNA from cultured cells was extracted using a G-spin™ Total DNA Extraction Mini Kit (Intron Biotechnology) according to the manufacturer’s instructions. Next, 300 ng of genomic DNA was amplified by touch-down PCR(36 cycles). Touch-down PCR conditions were as follows: 1 cycle at 98 °C for 30 s, followed by 22 cycles of 98 °C for 30 s, 72–60 °C for 30 s (a decrease of 1 °C every two cycles) and 72 °C for 1 min, followed by 14 additional cycles at 98 °C for 30 s, 60 °C for 30 s, and 72 °C for 1 min, and the final extension step at 72 °C for 10 min. For the second PCR, 0.5 μL (0.5 ~ 0.8 μg) of the touch-down PCR product was used. The second PCR conditions were as follows: 1 cycle at 98 °C for 30 s, followed by 35 cycles at 98 °C for 30 s, 65 °C for 30 s, and 72 °C for 1 min, with a final extension step at 72 °C for 10 min. The primers used for junctional PCR were as follows: (P1):TTTAGCCCCGGAATTGACTGGA, (P2):TCGTGGGGTCCAGGCCAAGTA, (P3):TGA GTCCGGACCACTTTGAGCT, (P4):AGGCTGACCCCAAATTCTCTGTAGG.

### Karyotyping

Karyotyping analysis was conduted at GenDix, Inc. (Seoul, Korea) using ChIPS-Karyo (Chromosome Image Processing System).

### Quantitative-reverse-transcriptase PCR and reverse-transcriptase PCR analyses

Quantitative (q) reverse-transcriptase (RT) PCR analysis was conducted as previously described [17]. Briefly, total RNA was isolated from cells using RNA-stat (Iso-Tex Diagnostics, Friendswood, TX, USA). Extracted RNA was reverse-transcribed using Taqman Reverse Transcription Reagents (Applied Biosystems, Foster City, CA, USA), according to the manufacturer’s instructions. The synthesized cDNA was subjected to qRT-PCR or RT-PCR using human-specific primers and probes. RNA levels were quantitatively assessed using the ABI PRISM 7000 Sequence Detection System (Applied Biosystems). Relative mRNA expression normalized to GAPDH expression was calculated as described previously [18]. The primers used for qRT-PCR were as follows: HGF (Hs00300159_m1), IL-10 (Hs00961622_m1) and GAPDH (Hs99999905_m1). The primers used for RT-PCR were as follows: 5′-TCAGCACTCGAAGGTCAAGC−/CACTCAACGAGAACCAGCAG-3′ for HNF4A (193 bp), 5′-CAATGCGGAAAGAGGGGATC−/CTGCTGTGCCCGTAGTGAGA-3′ for GATA4 (208 bp), 5′-GACTGGAGCAGCTACTATGCA−/CATGGGGCTCATGGAGTTCA-3′ for FOXA2 (357 bp), 5′-GAATGCCCTGTGCAGAAGAC−/ATGGAAGGTGAATGTTTCAGCA-3′ for ALB (197 bp), 5′-TCTTCACCACCATGGAGAAG−/CATGAGTCCTTCCACGATAC-3′ for GAPDH (224 bp).

### Induction of liver fibrosis and cell transplantation

All animal procedures were performed in accordance with the Guidelines for Care and Use of Laboratory Animals of Catholic Kwandong University and approved by the Animal Ethics Committee of Catholic Kwandong University. Six-week-old female BALB/c nu mice were purchased from Koatech (Pyeongtaek, Korea). The mice were intraperitoneally injected with 150 mg/kg thioacetamide (TAA; Sigma Chemicals, St Louis, MO, USA) two times a week for 4 weeks. After the induction of liver fibrosis (i.e., 1 week after TAA injection), cell transplantation was performed. Cells (1 × 10^6^) were resuspended in normal saline and administered via the portal vein. The mice were randomly divided into three groups: sham (PBS) (*n* = 10), iPS (*n* = 10) and iHep (*n* = 10). The sham group received 50 μl of normal saline. Liver tissues were collected 3 weeks after cell injection. We also confirmed that all experiments were performed in accordance with national guidelines and regulations.

### Histological analyses and collagen detection

The livers were fixed with 4% paraformaldehyde for 1 day and embedded in Tissue-Tek OCT compound (Sakura Fine Technical Co. Ltd., Japan). Sectioned (10 μm thick) liver tissues were stained with Harris hematoxylin solution (Sigma) for 2 min followed by eosin Y (Sigma, MO) for 20 s. Collagen and hepatic fibrosis were measured using a Sirius red/fast green collagen staining kit (Chondrex, Inc., Redmond, WA, USA. Sectioned samples were fixed in Kahle’s fixative for 30 min, and the sections were then stained in the dye solution for 30 min. The sections were rinsed with distilled water and incubated with a dye extraction buffer. Then, we collected the eluted dye solution and read the O.D. value at 540 nm and 620 nm with a spectrophotometer. Masson’s trichrome staining was performed, and the areas of fibrosis were measured by MetaMorph software (Downingtown, PA). Anti- proliferating cell nuclear antigen (PCNA,1:100, Abcam) was used for cell proliferation assay. TdT-mediated dUTP nick end labeling (TUNEL) assay was conducted using a fluorescein in situ cell death detection kit (Roche Molecular Biochemicals, Mannheim, Germany). Nuclei were stained with DAPI.

### Measurement of cytokine

Serum albumin content was examined using human albumin (ALB) ELISA kit (Ray Biotech, Norcross, USA) following the manufacturer’s instructions.

### Immunoblot analysis

Western blot assays were performed using a previously described method [19, 20]. Briefly, protein extracts (100 mg each) were separated on 8% SDS–PAGE gels (Bio-Rad Laboratories) and electro-transferred onto PVDF membranes (GE Healthcare). The specimens were probed with antibodies against the following: albumin (ALB) and β-actin (Santa Cruz Biotechnology, Inc.). The membranes were washed and incubated with horseradish peroxidase-conjugated secondary antibody, and the signal was detected using an LAS-3000 chemidoc system (Fujifilm, Japan).

### Statistical analysis

All data are presented as the mean ± SD. Statistical analysis was conducted by Student’s t-test (for two groups) and ANOVA with Bonferroni’s multiple comparison test using SPSS v11.0 (for comparison between multiple groups). *P* < 0.05 was considered statistically significant.

## Results

### Targeted knock-in of albumin-promoter (ALBpro) into the AAVS1 site

The entire process of obtaining induced hepatocyte-like cells (iHep) is described in Fig. 1a. First, iPS and HepG2 cell lines possessing ALB promoter (ALBpro, 844 bp) were established using TALEN-mediated integration. The donor plasmid was designed to carry the albumin promoter (ALBpro) and the GFP reporter gene (Fig. 1b). The transfected cells were selected through culturing with puromycin (49.3%) and differentiated into hepatocyte. GFP expressing cells were observed from the 7th day onwards (Fig. 1c).

### Confirmation of hepatic properties of the ALB::GFP expressing iHep cells

**Fig. 2:**
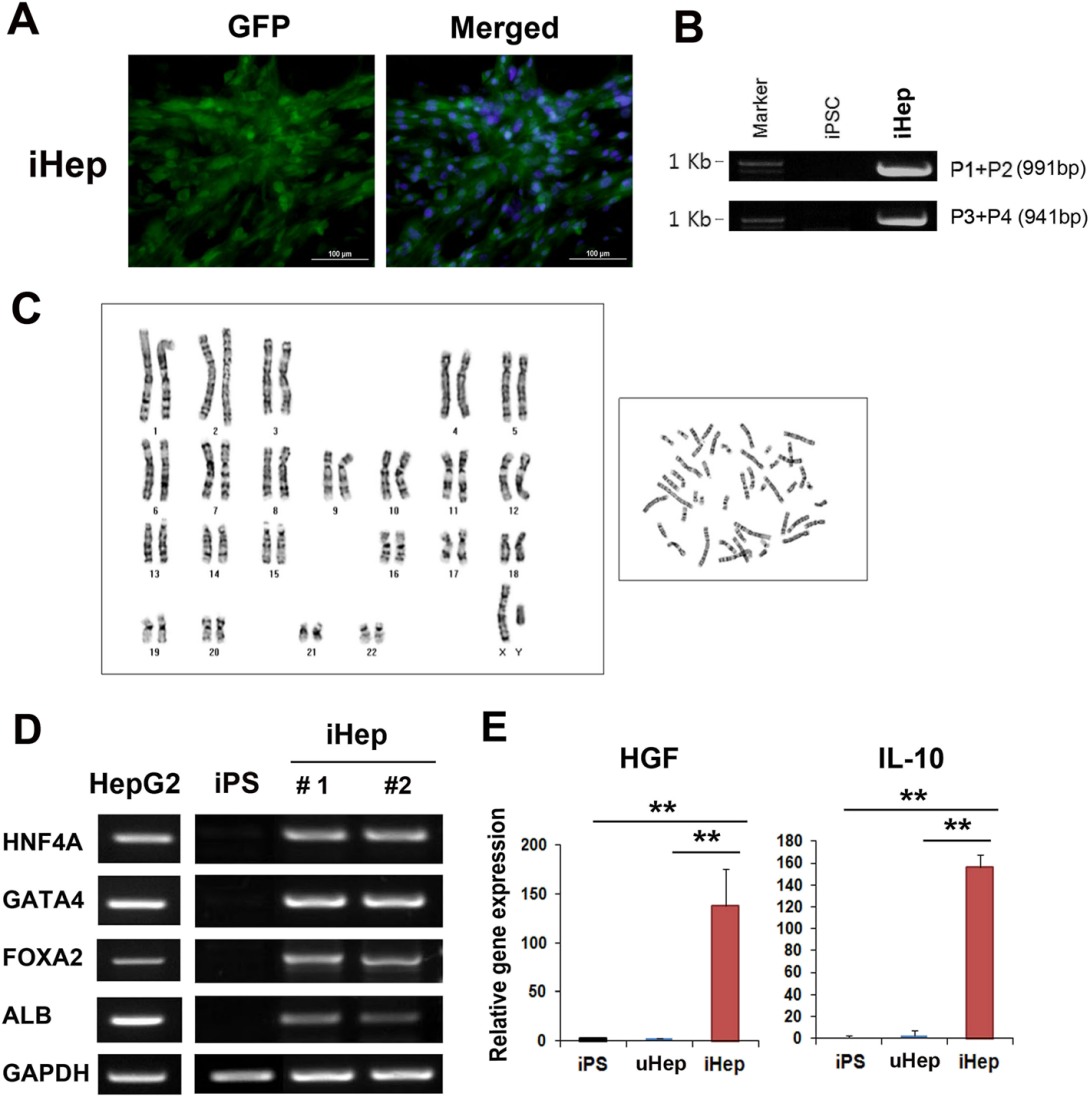
Hepatic properties of iHep cells. **a** Representative picture of FACS sorted pure iHep cells. Nuclei stained with DAPI (Blue color). Bar = 200 μm. **b** Correct insertion of donor plasmid into the AAVS1 locus was confirmed by junction PCR. Locations of primers for junction detection are indicated (primer F (P1, P3) and primer R (P2, P4)) in Fig. 1b. **c** Karyotype of iHep. **d** Hepatocyte specific gene expression in iHep cells. RT-PCR was performed using iHep 1 and 2 lines. **e** Hepatogenic and anti-fibrogenic properties of iHep cells. qRT-PCR was performed using iPS, uninduced ALBpro:iPS (uHep) and induced ALBpro:iPS (iHep)

**Fig. 3:**
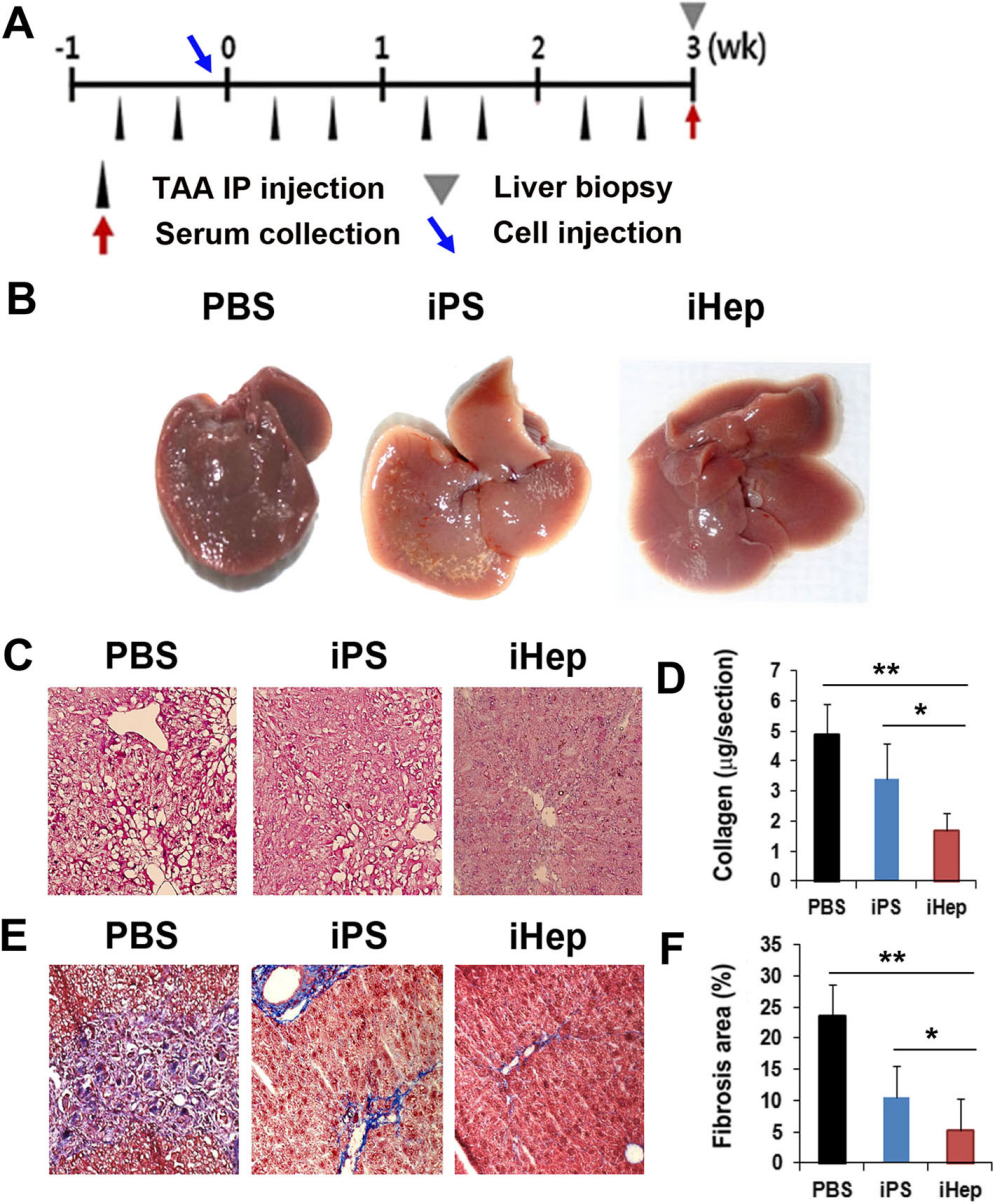
In vivo therapeutic effects of iHep cells in acute liver fibrosis. **a** A schematic diagram of the procedure for the in vivo study. **b** A representative picture of the damaged liver after iHep cell injection. The shrunken liver, irregular surface, and damaged fibrotic areas of the liver in the PBS- or iPS-treated mice were observed. **c** Representative pictures of H&E staining 3 weeks after cell transplantation. Bar = 100 μm. **d** Quantitative analysis of collagen content from liver tissues. The hepatic collagen content was measured using Sirius red/fast green collagen staining. Sectioned liver tissues were fixed in Kahle’s fixative and stained with dye solution and mixed with a dye extraction buffer. The O.D. values of dye solutions were read by a spectrophotometer. *n* = 7 per group. **p < 0.01; *p < 0.05. **e** Representative pictures of fibrosis by Masson’s trichrome (M-T) staining after cell transplantation. Bars = 100 μm. **f** Quantitative analysis of the fibrotic area by M-T staining. **p < 0.01; *p < 0.05, *n* = 10 per group

ALB::GFP expressing pure iHep cells were isolated using FACS sorter (99.8% GFP-positive cells) 7 to 10 days after hepatocyte differentiation process (Fig. 2a and Supplemetary Fig. 2). To confirm genomic integration of the donor plasmid into the AAVS1 site, we carried out genomic DNA PCR. Successful insertion of the donor plasmids into the iPS cell line was confirmed by the PCR product showing 5′ and 3′ junction fragment amplification (Fig. 2b). In addition, iHep displayed normal karyotype (Fig. 2c). Next, to confirm the reporter system is functional, we measured the expression level of ALB protein in iHep using Western blot. iHep cells highly expressed ALB protein compare with the iPS or uHep (Supplemetary Fig. 3).

We also analyzed the hepatocyte-specific gene expression patterns by RT-PCR to confirm the hepatic properties of these iHep cells. The results demonstrated that 4 representative hepatocyte-specific genes such as HNF4a, GATA4, FAXA2, and ALB were upregulated in the iHep cells as compared to the control iPS (Fig. 2c). Next, we evaluated the liver function recovery capacity of the iHep cells. The expression levels of two representative anti-inflammatory factors, HGF and IL-10, were evaluated. Interestingly, the iHep cells exhibited significantly high expression levels of HGF and IL-10 as compared to iPS or uninduced iHep cells (uHep) (Fig. 2d).

### Morphological evidence of therapeutic potential of iHep cells

The therapeutic effect of iHep cells in cases of acute liver fibrosis was assessed by in vivo transplantation of 1 × 10^6^ iHep cells into mice via an injection in the portal vein 1 week after TAA-induced acute liver damage. The liver tissues were harvested 3 weeks after the cell transplantation (Fig. 3a). Morphological analysis of the PBS and iPS treated liver tissues revealed extensive scarring, while the iHep treated liver tissues exhibited relatively healthy morphology (Fig. 3b).

Next, to evaluate the anti-fibrotic property of iHep, we performed hematoxylin and eosin (H&E) and Masson’s trichrome (M-T) staining on each group of liver tissues. H&E staining revealed that the PBS- and iPS-treated liver tissues displayed a distorted hepatic architecture as compared to the iHep treated liver tissues (Fig. 3c). We also assessed the hepatic collagen content in the liver tissues after cell transplantation. The hepatic collagen content was measured using Sirius red/fast green collagen staining. Sectioned liver tissues were fixed in Kahle’s fixative and stained with dye solution and mixed with a dye extraction buffer. The O.D. values of dye solutions were read by a spectrophotometer. The hepatic collagen content of the iHep treated liver tissues was significantly lower than that of the PBS- or iPS-treated liver tissues (Fig. 3d), indicating an active extracellular matrix (ECM) remodeling. M-T staining showed that iHep treatment significantly reduced liver fibrosis as compared to that in the PBS- or iPS-treated group (Fig. 3e and f).

### Biochemical evidence of therapeutic potential of iHep cells

**Fig. 4:**
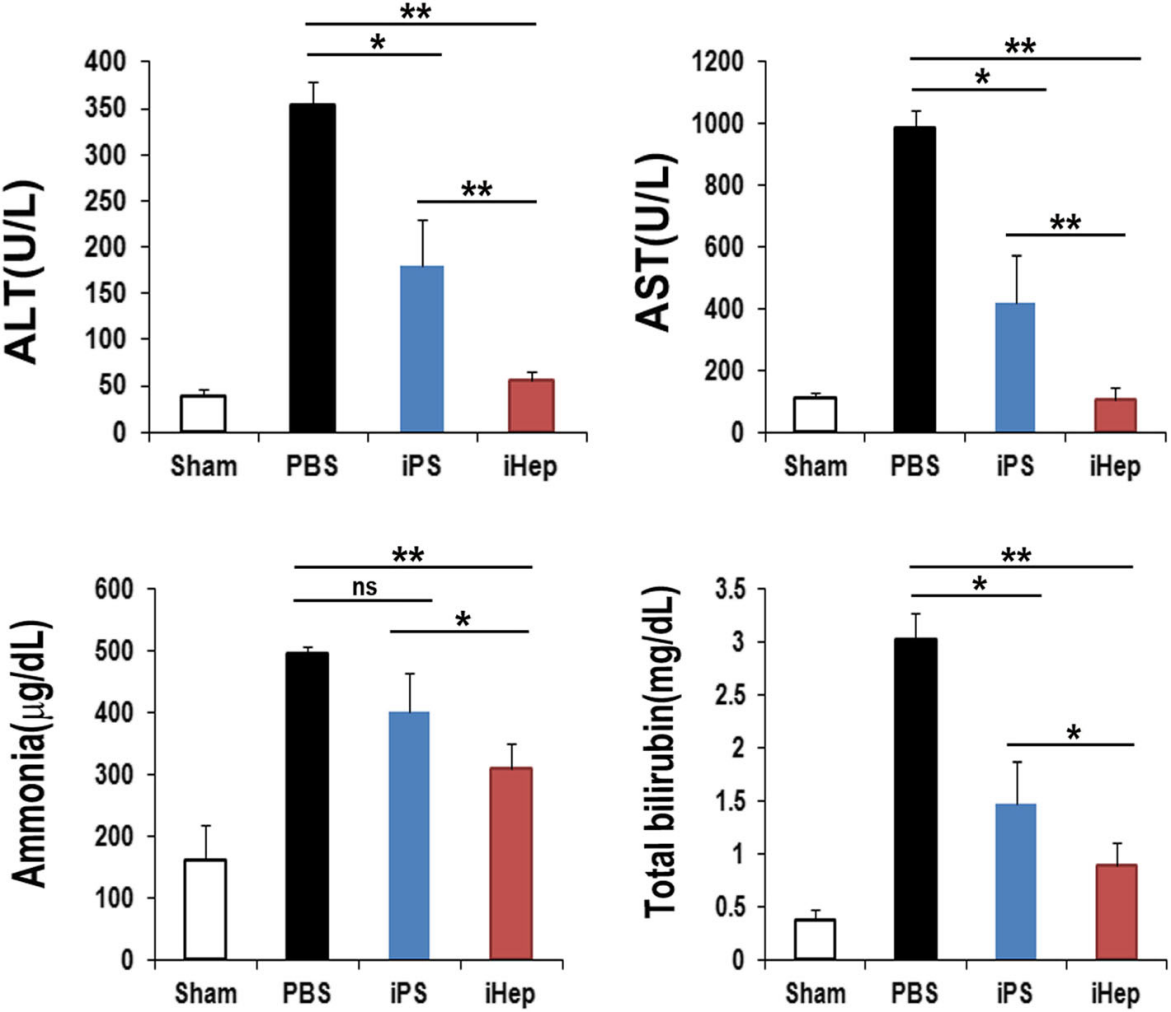
Biochemical analysis of serum after iHep cell injection. Concentrations of ALT, AST, ammonia, and total bilirubin in the serum of mice 3 weeks after cell transplantation. **p < 0.01; *p < 0.05; *n* = 10 per group

Biochemical parameters were analyzed using mouse serum 3 weeks after iHep cell transplantation to assess the therapeutic potential of the iHep cells in damaged liver. The levels of alanine aminotransferase (ALT), aspartate aminotransferase (AST), ammonia, and total bilirubin were significantly decreased in the iHep-treated mice as compared to the PBS or iPS-treated mice (Fig. 4). These results suggest that iHep cell transplantation is clearly beneficial for the functional recovery of damaged liver.

### Elucidation of regeneration mechanisms of transplanted iHep cells

**Fig. 5:**
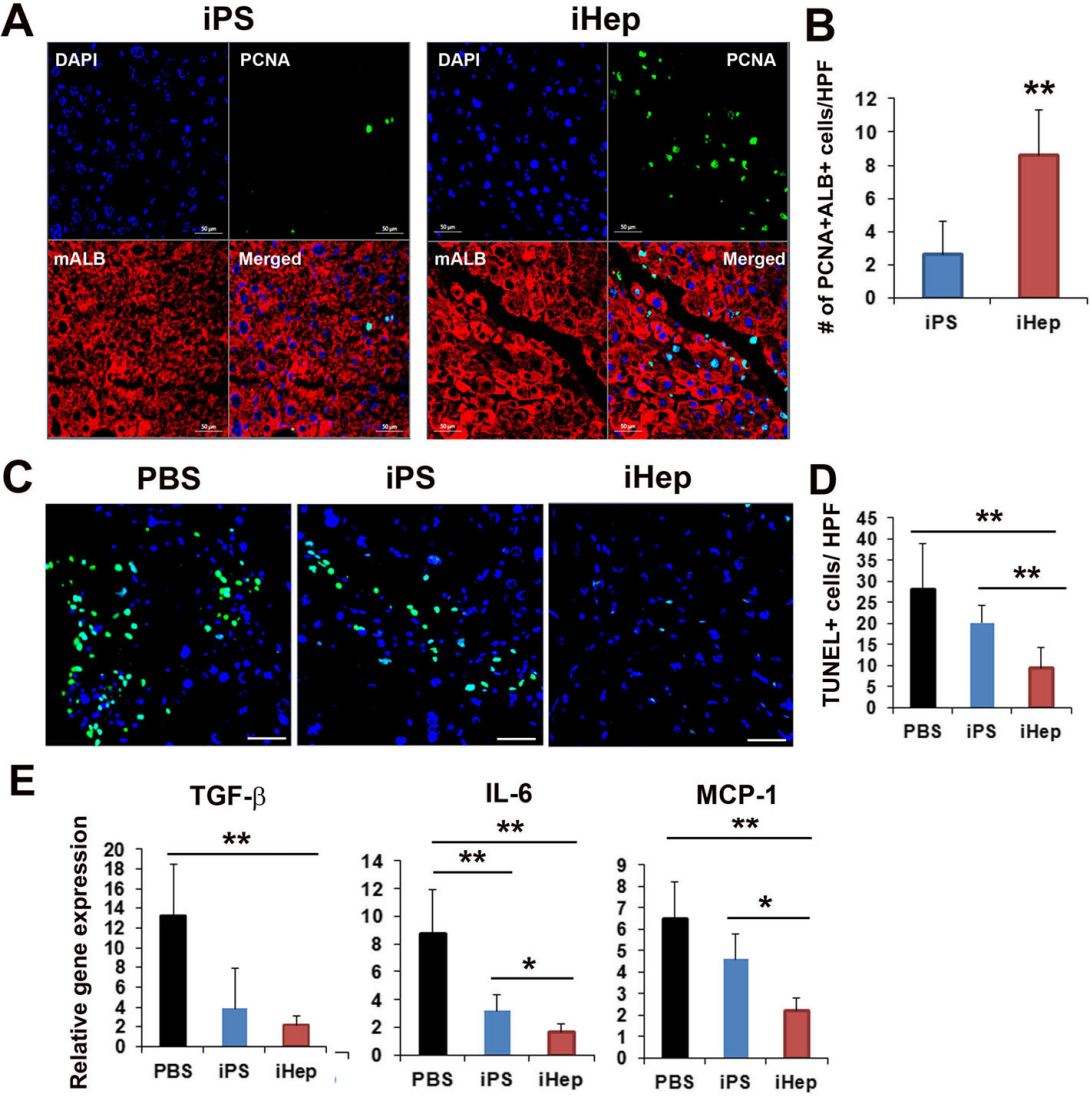
Evidence of liver regeneration after iHep cell transplantation. **a** Representative images of PCNA (green) and anti-mouse albumin (red)-positive hepatocytes in liver tissues by immunohistochemistry. Bar = 20 μm. **b** Quantitative analysis of PCNA staining. **p < 0.01; *n* = 7 per group. **c** Representative images of TUNEL (green) and DAPI (blue)-positive cells in liver tissues by immunohistochemistry. Bar = 20 μm. **d** Pro-inflammatory gene expression in liver tissue after cell injection evaluated by qRT-PCR. **p < 0.01; *n* = 7 per group. Abbreviation: HPF, high power field

To elucidate the mechanisms underlying amelioration of liver function after iHep cell injection, the proliferation of hepatocytes in the liver was evaluated. Because iHep express high level of HGF. Histological observations showed significantly higher numbers of PCNA-positive hepatocytes in iHep-injected liver tissues than in iPS- or in PBS-injected liver tissues (Fig. 5a and b).

In addition, we measured the number of apoptotic hepatocytes using terminal deoxynucleotidyl transferase dUTP nick end labeling (TUNEL). Expectedly, lower number of TUNEL positive hepatocytes were observed in iHep-treated liver tissues as compared to PBS- or iPS-treated liver tissues (Fig. 5c and d).

Next, to evaluate the pro-inflammatory mechanisms, we performed the qRT-PCR using liver tissues 3 weeks after the iHep cell injection. Interestingly, the expression levels of transforming growth factor (TGF)-β, interleukin (IL)-6, and monocyte chemo attractant protein (MCP)-1 were significantly down-regulated in iHep injected liver tissues as compared to iPS or PBS injected liver tissues.

### Analysis of fate and functionality of engrafted iHep cells

**Fig. 6:**
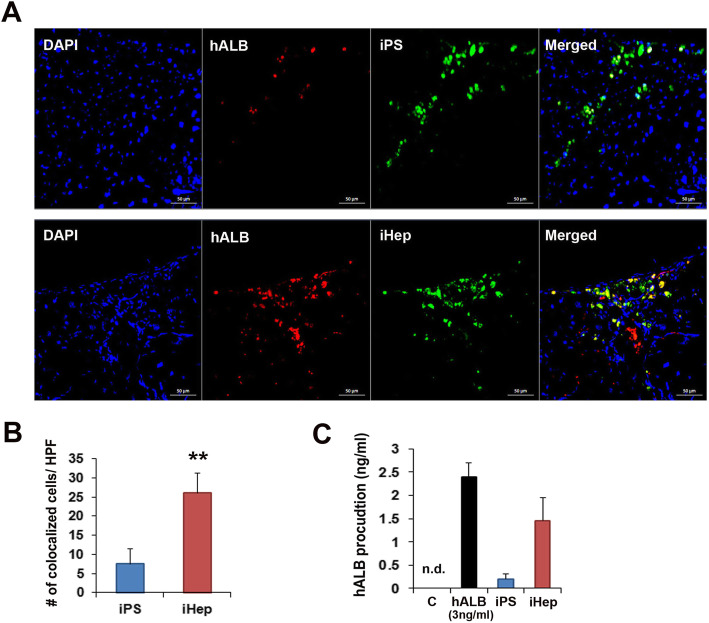
hALB-expressing iHep cells engrafted in the liver tissue. **a** Representative images of hALB expressing iHep cells after cell transplantation in the fibrotic liver. **b** Quantitative analysis of hALB-expressing cells in the liver. **p < 0.01; *n* = 6 per group. **c** hALB production in mice serum after iHep cell injection. hALB was assayed using ELISA at 3 weeks after cell injection. *n* = 5 per group. Abbreviation: ND, not determined

Histological analysis of the periportal and perisinusoidal regions was performed using human albumin (hALB) marker to trace the fate of the injected iHep cells. Higher number of hALB expressing iHep cells was observed engrafted in the periportal and the perisinusoidal regions as compared to iPS cells (Fig. 6a and b).

Next, to confirm the functionality of the engrafted iHep cells in mice liver, we performed an ELISA analysis of mice serum for the detection of hALB. While the serum of PBS- or iPS-treated mice showed no cross-reaction, hALB was detected (1.48 ± 0.52) in the serum of iHep-treated mice (Fig. 6c).

## Discussion

The present study intended to prove the therapeutic potential of induced hepatocyte-like cells (iHep) when used as an alternative to liver transplantation in cases of end-stage liver disease and fulminant liver failure. Therefore, we first generated ALB-GFP hepatocyte-reporter cells derived from iPS using genome editing. These transgenic iHep cells showed hepatic properties such as functional hepatocyte activity in vitro and in vivo. These pure iHep cell lines will provide a novel research alternative for drug discovery or cell therapy applications.

Recently, induced pluripotent stem (iPS) cells have been developed for the generation of tissue-specific cell types [21–23]. However, their low differentiation capacity and slow speed of differentiation have proved to be limiting factors. The iPS derived differentiated cell lines show heterogeneity which further restricts their use. These heterogenic cells could cause uncontrolled proliferation, which is a serious concern threatening the safety of their usage in vivo. To overcome this hurdle, we generated ALB::GFP iPS reporter cell lines (iHep) using the TALEN system. TALEN is an efficient and reliable technology for targeted gene editing. We used the AAVS1 locus on chromosome 19 for the specific targeting owing to its proved efficiency at harboring such gene insertions.

Once the reporter system integrated iPS cell lines were established and differentiated into iHep cells, these were isolated using ALB promoter. ALB expression in the liver is well characterized and has been reported in hepatocytes and most livers at embryonic and adult stages [24]. The growth factor-induced hepatocyte differentiation protocol enabled generation of iHep cells after an incubation period of 10–15 days. A total of 99% of iHep cells sorted using FACS showed GFP-positivity, thereby confirming a homogenous hepatocyte population. Therefore, we speculate that ALB::GFP iHep cells can provide a suitable model to study drug development in vitro or treat human hepatic diseases in vivo.

The isolated iHep cells were characterized for morphological, biochemical, and immunological parameters. Interestingly, the iHep cells showed high levels of HGF and IL-10 expression. Certain humoral factors present in stem cells or progenitor cells are known to ameliorate acute or chronic liver failure [25]. It is known that HGF regulates cell growth and migration by activating a tyrosine kinase signaling pathway through the c-Met receptor and plays a key role in angiogenesis and the development and regeneration of livers [26, 27]. A study has also shown that administration of HGF in cases of liver injury promotes the expression level of ALB and increases the survival rate of mice [28]. Many studies have demonstrated that HGF exerts an important role in angiogenesis and hepatocyte differentiation from iPS. This study showed that transplantation of iHep cells into severely damaged liver tissues exerts anti-fibrotic effects and improves the function of the damaged liver. We also elucidated the therapeutic mechanisms of the transplanted iHep cells. Interestingly, high numbers of PCNA-expressing hepatocytes and low numbers of TUNEL-expressing hepatocytes were observed in the iHep-injected liver tissues. These results indicate that the humoral factors present in iHep cells accelerate liver regeneration by decreasing apoptotic hepatocyte count. HGF secreted from iHep cells may be particularly responsible for positively influencing these favorable changes in the damaged liver tissue. We traced the fate of the injected iHep cells in vivo and found high concentrations of ALB expressing iHep cells in the perivascular space of the liver tissue. The high concentrations of the engrafted iHep cells in the liver could also be an important contributing factor in liver regeneration. These results in summary indicate that iHep cells may possess multiple therapeutic abilities to promote liver function in a damaged liver.

IL-10 is widely known as a representative anti-inflammatory factor that regulates pro-inflammatory mediators contributing to liver fibrosis [29, 30]. IL-10 is also known to play a key role in liver fibrosis by promoting apoptosis of hepatic satellite cells (HSCs) and regulating their function [31, 32]. IL-10 also modulates the production of collagen and collagenases, thereby affecting extracellular matrix remodeling [33]. In fact, the administration of IL-10 has been shown to decrease inflammatory factors and suppress the apoptosis of hepatocytes [34, 35]. In line with these reports, transplantation of IL-10 expressing iHep cells was seen to attenuate the expression of pro-inflammatory cytokines TGF-β, IL-6, and MCP-1 in the liver tissues. These results suggest that IL-10 delivery might promote anti-inflammatory function and enhance anti-fibrotic activity.

In conclusion, the generation and use of pure hepatic iHep cells might provide a novel tool for drug screening/discovery, studying the mechanism of action or cell therapy for treating liver fibrotic diseases. However, a more sophisticated cell differentiation protocol for the generation of more specific hepatic cells from iPS would be beneficial.

## Supplementary information

**Additional file 1: Supplementary Figure 1.** TALEN vector information (System Biosciences). (A) AAVS1 target sequence. (B) AAVS1 TALEN pair vector (C) AAVS1 donor vector. **Supplementary Figure 2.** FACS sorting results. (A) Gating strategy to isolate GFP+ iHep cells. (B) The rate of GFP+ cells after sorting. **Supplementary Figure 3.** Western blot analysis. The expression of ALB protein was detected in iHep. uninduced ALBpro:iPS (uHep).

## Data Availability

Data sharing is not applicable to this article as no datasets were generated or analyzed during the current study.

## Abbreviations

ALB: Albumin
ALBpro: Albumin-promoter
DAPI: 4′-6-diamidino-2-phenylindole
H&E: Hematoxylin and eosin
HGF: Hepatocyte growth factor
iHep: induced hepatocyte-like cells
iPSCs: induced pluripotent stem cells
IL-10: Interleukin − 10
M-T: Masson’s trichrome
MCP-1: Monocyte chemo attractant protein
TAA: Thioacetamide
TALEN: Transcription activator-like effector nucleases
TGF-β: Transforming growth factor-β
