# Supplementary material for: HGF and IL-10 expressing ALB::GFP reporter cells generated from iPSCs show robust anti-fibrotic property in acute fibrotic liver model
Source: Stem Cell Res Ther. 2020 Aug 3;11:332. doi: 10.1186/s13287-020-01745-0 (PMC7398392; doi:10.1186/s13287-020-01745-0)

**Supplementary Data**

**Supplementary Figure Legend**

**Supplementary Figure 1**. TALEN vector information (System Biosciences). (A) AAVS1 target sequence. (B) AAVS1 TALEN pair vector (C) AAVS1 donor vector.

**Supplementary Figure 2.** FACS sorting results. (A) Gating strategy to isolate GFP+ iHep cells. (B) The rate of GFP+ cells after sorting.

**Supplementary Figure 3.** Western blot analysis. The expression of ALB protein was detected in iHep. uninduced ALBpro:iPS (uHep).

**Supplementary Figure 1.**

**
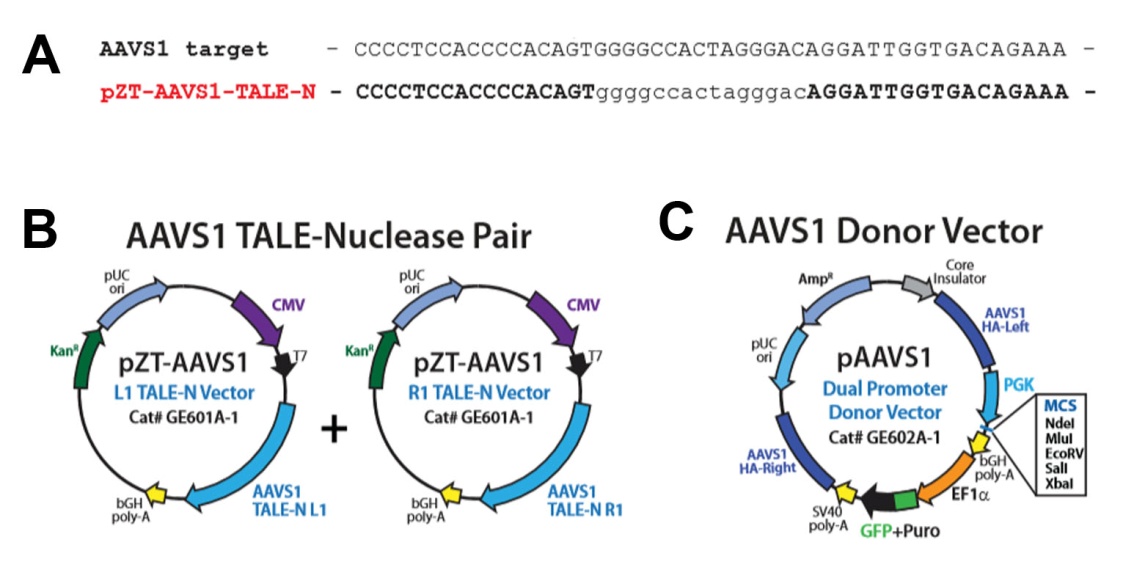
**

**Supplementary Figure 2.**


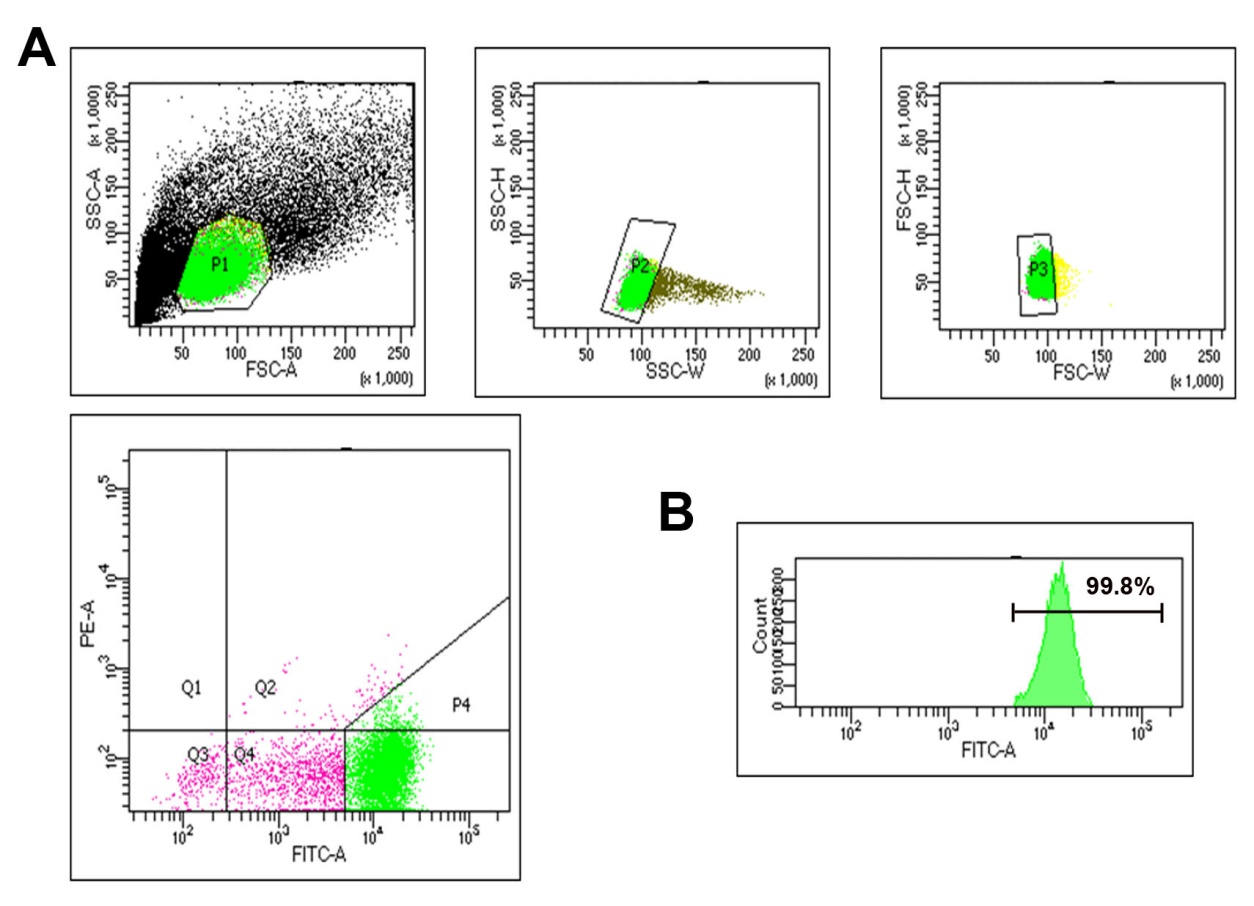


**Supplementary Figure 3.**


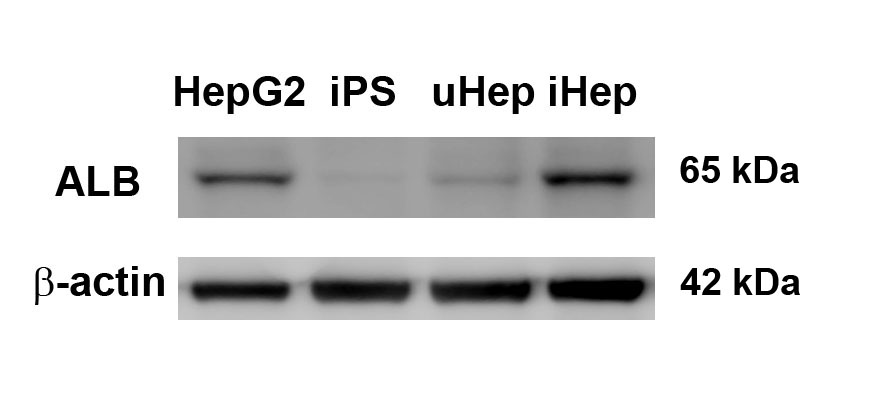

Supplement: Supplementary file 1 — Additional file 1: Supplementary Figure 1. TALEN vector information (System Biosciences). (A) AAVS1 target sequence. (B) AAVS1 TALEN pair vector (C) AAVS1 donor vector. Supplementary Figure 2. FACS sorting results. (A) Gating strategy to isolate GFP+ iHep cells. (B) The rate of GFP+ cells after sorting. Supplementary Figure 3. Western blot analysis. The expression of ALB protein was detected in iHep. uninduced ALBpro:iPS (uHep). [file 13287_2020_1745_MOESM1_ESM.docx]
